# Sleep Spindles as Facilitators of Memory Formation and Learning

**DOI:** 10.1155/2016/1796715

**Published:** 2016-03-28

**Authors:** Daniel Ulrich

**Affiliations:** Department of Physiology and Institute of Neuroscience, Trinity College Dublin, Dublin, Ireland

## Abstract

Over the past decades important progress has been made in understanding the mechanisms of sleep spindle generation. At the same time a physiological role of sleep spindles is starting to be revealed. Behavioural studies in humans and animals have found significant correlations between the recall performance in different learning tasks and the amount of sleep spindles in the intervening sleep. Concomitant neurophysiological experiments showed a close relationship between sleep spindles and other sleep related EEG rhythms as well as a relationship between sleep spindles and synaptic plasticity. Together, there is growing evidence from several disciplines in neuroscience for a participation of sleep spindles in memory formation and learning.

## 1. Introduction

**Figure 1:**
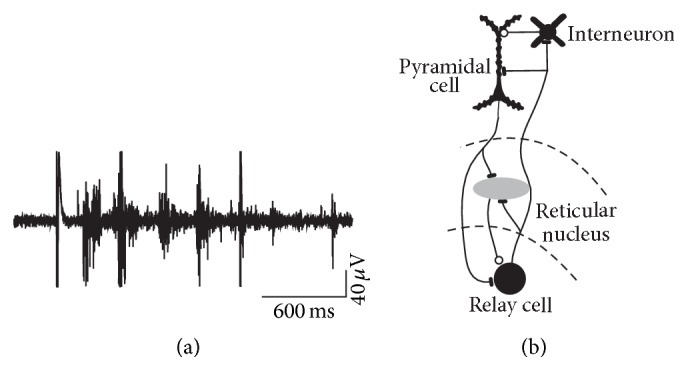
(a) Spindle discharges recorded in thalamus with extracellular electrodes. (b) Schematic diagram of the main cell types in thalamus and cortex participating in spindles. Thalamocortical relay cells excite inhibitory neurons of the adjacent reticular nucleus. Relay cells in turn are reexcited via postinhibitory rebound from reticular neurons. Thalamus and cortex are reciprocally connected by axons of relay cells and pyramidal neurons.

Sleep spindles are waxing and waning 7–14 Hz EEG rhythms that occur during various stages of non-REM sleep (Figure 1(a)). They are generated in the thalamus through alternating excitation of relay cells and reticular neurons (Figure 1(b)). Spindles are propagated from thalamus to the cortex by thalamocortical axons and get synchronized via corticothalamic projections [1]. Spindles were originally thought to occur at sleep onset but were subsequently shown to be generated throughout sleep, particularly in association with slow oscillations. Although mechanistically not fully understood faster centroparietal spindles in humans are distinguishable from slower prefrontal spindles [1]. There is support from behavioural, physiological, and cellular studies for an active role of sleep in learning. According to two separate conceptual frameworks, the two major sleep episodes, non-REM and REM sleep, contribute sequentially or in parallel to sleep-dependent memory formation. In the dual model, non-REM sleep predominantly facilitates declarative learning, that is, the memories for facts and events, while REM sleep affects mainly procedural learning [2]. Alternatively, in the sequential model all types of memories are shaped by the alternating occurrence of non-REM and REM sleep episodes [3]. In this review I discuss behavioural and neurophysiological data that support a role of sleep spindles in memory formation summarizing recent findings over the past decade. For a more general discussion of the role of sleep in learning, the reader is referred to recent comprehensive review articles [4–8].

## 2. Sleep Spindles and Learning

Combined behavioural and electroencephalographic studies mainly on humans but also on experimental animals show an association between sleep spindle activity and performance in different learning paradigms. In a word-pair association task, an increased recall performance was found in people with high spindle activity during the intervening night involving slow as well as fast spindles [9]. Spindle activity was quantified by combining measurements of spindle amplitude and duration. The conclusion of this study was that the occurrence of sleep spindles had a beneficial influence on memory formation. In a similar experimental setup, an increase in spindle density was recorded after a word-association task [10]. In addition, in these latter experiments spindle density correlated linearly with recall performance. A similar linear relationship was found between spindle number and a lexical competition parameter that measures the integration of new words into the existing vocabulary indicating that spindles can play a role in the processing of novel information [11]. A comparable beneficial role of slow sleep spindles was seen in a word-pair learning task involving a daytime nap [12]. This latter finding largely excludes a major contribution of circadian rhythms that are known to influence sleep spindle activity and learning [13]. In addition, Schmidt et al. [12] showed that the role of slow spindles became significant only above a certain level of complexity of the task. It was subsequently shown that a linear correlation between spindle density and recall performance was not ubiquitous as it applied to verbal learning and visuospatial memory but not to a facial recognition test [14, 15]. Spindles occur during various stages of non-REM sleep and can be associated with slow oscillations during slow-wave sleep. In a visual learning task, Cox et al. found that the sleep spindles particularly occurring during slow-wave sleep are responsible for potentiating memories [16]. However, in another study involving procedural motor learning, an increase in spindles was associated with stage 2 sleep, that is, light sleep [17]. It is thus possible that in humans different learning paradigms benefit from either type of spindles (slow or fast) during different phases of non-REM sleep.

A series of complementary studies investigated the role of sleep spindles in procedural learning in humans. Morin et al. [18] found increased spindle activity after a motor sequence learning tasks. A correlation between motor sequence learning and spindle activity was also found during daytime naps, again rendering a significant contribution of circadian factors rather unlikely [19].

Neurological and psychiatric conditions like Alzheimer's disease and schizophrenia are associated with decreased memory performance and reduced spindle activity during sleep [20]. Similarly, a decline in learning capabilities in the elderly correlates with diminished sleep spindle activity in prefrontal cortical areas [21]. These examples complement the studies in healthy subjects by demonstrating a relationship between spindles and memory also under conditions of decreased performance.

A comparable increase in sleep spindle density was found in laboratory rats trained in an odor-reward association task [22]. In addition, increased spindle density was also associated with retrieval of remote memories and memory updates (reward extinction) indicating that an involvement of sleep spindles in learning is not confined to sleep episodes immediately after training [22]. In the same species, spindles particularly at the transition to REM sleep were shown to be involved in the consolidation of novel memories [23]. These studies show that spindles play a role in learning also in other species and that laboratory animals can be used to investigate the underlying mechanisms (see below).

The correlative relationship of sleep spindles and memory task performance is suggestive of additional experiments involving selective manipulations of spindle activity to further support an active role of spindles in memory formation. Enhanced spindle activity during non-REM sleep induced by transcranial alternating current stimulations in humans leads to increased recall performance in a declarative word-association task [24]. In that study, spindle waves and slow oscillations were both enhanced compared to controls who received only sham stimuli. Similarly, pharmacological augmentation of spindles with the allosteric GABA_A_ receptor modulator Zolpidem increased the recall performance in a study involving a verbal memory task [25]. However, in the same study, application of the hypnotic sodium oxybate, which is known to decrease spindle activity, had no negative effect on memory recall. Nevertheless, a reduced performance in a declarative but not procedural memory task could be observed after decreasing spindle power during non-REM sleep by theta frequency transcranial stimulation [26]. Increased spindle activity induced by selective uptake blockers for serotonin or norepinephrine in humans also improved procedural memory as assessed by a finger sequence tapping and mirror tracing task [27]. The non-REM sleep related improvement in the finger tapping sequence task was again abolished after decreasing spindle power with the GABA reuptake blocker Tiagabine [28]. A recent study used an optogenetic approach to suppress spindle activity in mice by silencing inhibitory neurons of the thalamic reticular nucleus [29]. This manipulation was associated with a decreased performance of the animals in a novel object recognition task indicating that learning deficits can be induced by direct interference with the sleep spindle generating neural network.

Supplementary evidence for a role of spindles in memory formation was recently provided by the finding that learning related changes in spindle activity occur preferentially in task-associated areas of the brain. Nishida and Walker showed increased spindle activity in the contralateral hemisphere after a unilateral finger tapping task [19]. Similarly, by using standardized low-resolution brain electromagnetic tomography, Tamaki et al. [30] found a localized increase in sleep spindles after visuomotor learning in brain areas that are known to be involved in the task. A regional increase in spindles was also observed in electrocorticographic recordings in patients who were trained on a brain-computer interface [31]. In addition, that study showed that the increased spindle activity was due to an increased gain in existing spindle networks rather than due to the formation of novel ones. A recent study used memory cueing to investigate the role of spindles in memory formation [32]. These authors paired a word-location task with particular odors. During subsequent sleep, topographically localized spindles could be evoked by the corresponding odorant.

Together, a variety of studies show comprehensive correlative data for a role of sleep spindles in declarative and procedural memory consolidation in humans and laboratory animals in task relevant brain areas. In addition, memory performance can be up- or downregulated by experimentally enhancing or decreasing spindle activity.

## 3. Mechanisms of Sleep Spindle Associated Learning

Learning is thought to result from modifications of synaptic connections. In particular, long-term potentiation (LTP), a long-lasting enhancement of synaptic strength between excitatory neurons, has been widely investigated and is considered to be a key process in memory formation [33]. However, the inverse process of long-term synaptic depression was also shown to contribute to learning [34]. In addition, similar plasticity processes have been shown to occur at inhibitory synaptic contacts and between excitatory and inhibitory neurons [35]. It is thus likely that learning involves modifications at different types of synapses involving strengthening and weakening of subsets of neural connections. The two main paradigms regarding the role of sleep in learning are the synaptic downscaling and the systems consolidation hypotheses [4, 6]. According to the former, non-REM sleep leads to weakening of synaptic connections as part of a homeostatic process while in systems consolidation synapses are strengthened via replay of wake-related neural activity within and between different brain areas, in particular the hippocampus and neocortex [36, 37]. LTP is the most intensively studied form of synaptic plasticity and is known to proceed through different stages (early, late) eventually leading to long-lasting enhancement of synaptic transmission [38]. A key regulator of synaptic modifications is the secondary messenger Ca^2+^ [39]. A combination of parameters like ion concentration and kinetics seem to determine the sign and size of changes in synaptic strength. In the following, experimental evidence of the role of spindles in synaptic plasticity will be discussed.

Sejnowski and Destexhe [40] hypothesized that sleep spindles may contribute to learning by triggering calcium influx into pyramidal cells of neocortex that then induces the relevant plasticity processes. Indeed, spontaneous spindles in cats led to an increased responsiveness of neurons to synaptic stimuli [41] indicating that spindles enhance postsynaptic responsiveness to synaptic inputs. However, the spindle induced depolarizations may be restricted to dendritic compartments of neocortical pyramidal cells as concomitant spindle associated synaptic inhibition may prevent these depolarizations from reaching the soma and hence induce strong spike firing. This scenario would be in line with the relatively low firing frequency of pyramidal cells in neocortex during spindles that were observed with electrophysiological techniques in cats [42]. The model is further supported by experimental observations of dendritic Ca^2+^ influx during inhibition of the soma via GABA receptor activation [43] and the prevalence of inhibitory cell activity during spindles in naturally sleeping rats as seen with extracellular tetrode recordings [44].

**Figure 2:**
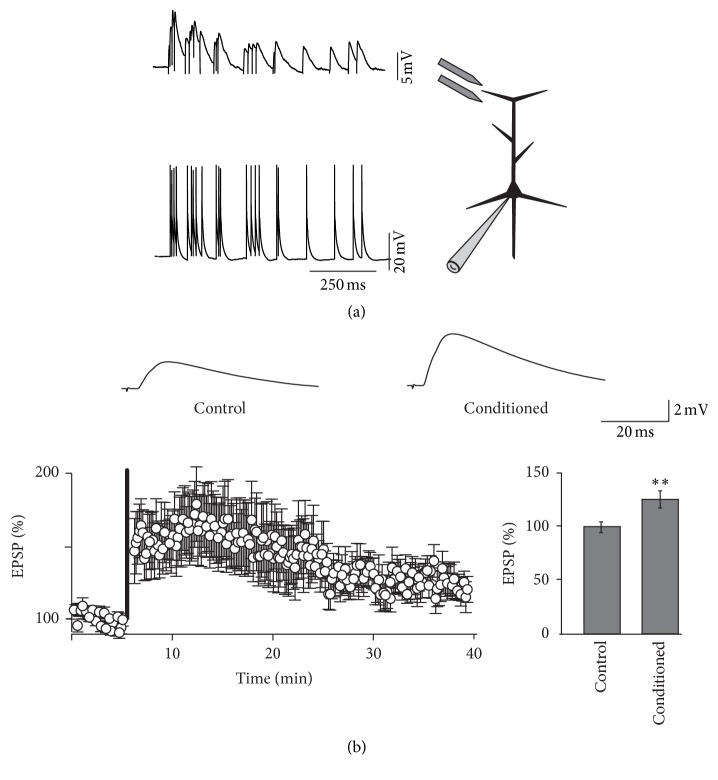
(a) Experimental approach for studying the role of spindles in synaptic plasticity. Spindle discharge patterns were used to trigger excitatory postsynaptic potentials (EPSPs) in pyramidal neurons. The same spindle trains were used to elicit action potentials in the soma. (b) Sample traces and EPSP time course before and after spindle conditioning. Concurrent pre- and postsynaptic spindles lead to statistically significant long-term synaptic potentiation (*∗∗*).

While spindles may be spontaneously generated, electrical stimulation of excitatory cortical synapses led to an enhanced reliability of spindles in rats [45]. Similarly, paired associative stimulation of the median nerve with transmagnetic stimulation of the contralateral motor cortex led to increased spindle activity in humans [46]. Both findings suggest that spindles can be preferentially triggered in networks that were active in the recent past. In particular, synchronous pre- and postsynaptic spindle-like activity in rat cortex in vitro was shown to induce long-term potentiation of excitatory postsynaptic potentials (Figure 2) while asynchronous spindles induced either no change or a mild synaptic depression [47]. Latter result may indicate that the impact of spindles on synaptic strength may depend on the relative occurrence of pre- and postsynaptic events similar to temporal contiguity requirements at hippocampal synapses [48]. Indeed in an elegant recent study using closed loop stimulation, Ngo et al. [49] found increased declarative memory consolidation for auditory stimuli applied in phase and not out-of-phase with slow-wave rhythms including spindles. Because different cell types in neocortex discharge action potentials at different phases relative to an ongoing spindle [44, 50] and individual neurons within a given class can spike at variable delays during spindling [51], there is a range of relative spike timings in the activated network relative to a given input that would lead to a differential adjustment of synaptic weights. In agreement, sleep-dependent ocular dominance shifts after monocular deprivation were shown to be associated with characteristic alterations in the spike field coherence of inhibitory and excitatory neurons relative to sleep spindles [52] probably reflecting modifications of their synaptic inputs. Spike-order dependent synaptic plasticity may also be involved in the hippocampal-neocortical replay during which hippocampal sharp waves precede thalamocortical spindles by a few dozen milliseconds [53].

Various mechanisms have been postulated to account for strengthening or weakening of synaptic connections. In particular long-term potentiation at excitatory synapses was shown to be associated with the growth of synaptic spines [54]. Interestingly in a recent report, Yang et al. [55] showed a non-REM sleep-dependent increase in dendritic spines associated with increased performance in a motor learning task in mice. This finding is all the more remarkable as the overall spine number was previously shown to decrease during sleep [56]. It is thus conceivable that sleep associated learning is at least partially mediated by the formation of new dendritic spines. Spine growth can be promoted by dendritic Ca^2+^ spikes [57] and L-type Ca^2+^ channels are known to contribute to those dendritic spikes [58]. In agreement, LTP of excitatory postsynaptic potentials induced by spindle trains in slices depended on influx of Ca^2+^ via L-type Ca^2+^ channels [47]. In addition to their role as charge carriers L-type channels are known to be specifically linked to excitation-transcription coupling related to inter alia the formation of new dendritic spines [59–61]. Sleep spindles may thus be responsible for transforming primary synaptic plasticity processes into more permanent forms by promoting structural plasticity.

## 4. Conclusions and Outlook

In conclusion many studies show a relationship between spindle activity and learning. In addition the topographically restricted modulation of spindles indicates their relation to ongoing brain activity. More importantly, selective manipulations that increase or decrease spindle activity lead to a corresponding improvement or deterioration of learning. Further studies are needed to clarify more precisely the role of spindles in learning. For example, a recent report suggests that sleep spindles are reflecting the overall learning capabilities rather than sleep-dependent memory formation [62]. Spindles are known to vary with age, gender, and measures of intelligence. How do those factors affect the quantitative relationships between spindles and learning and how do they contribute to the apparent diversity in published findings? What exactly is the quantitative relationship between spindle incidences and memory performance and which spindle characteristics (frequency, density, amplitude, and/or duration) are of relevance? This issue is of interest in light of differential ontogenetic changes of those parameters [63]. How can an apparent differential role of slow and fast spindles for different memory tasks be reconciled with a uniform mechanism? Why are spindles peculiar? This question suggests itself in view of the observation that birds, the other animal order with alternating non-REM and REM sleep episodes and sleep-facilitated learning, do not disclose sleep spindles [64]. What are the consequences of interactions of spindles with other brain oscillations, in particular gamma rhythms for synaptic plasticity?

Little is known about the cellular mechanisms of spindle associated memory formation. The observed phase shifts in spike firing in different cell types during spindles are possibly the consequence of synaptic plasticity processes. The close temporal associations of hippocampal ripples and spindles are a paradigm for NREM sleep based memory formation and may induce plasticity processes related to spike-timing dependent or heterosynaptic plasticity phenomena. The postulated spindle associated dendritic Ca^2+^ influx in combination with the known relationship between dendritic Ca^2+^ spikes, spine formation, and learning suggests a role for spindles in spine growth, a hypothesis that needs future experimental investigations.
